# Can Positron Emission Tomography and Computed Tomography Be a Substitute for Bone Marrow Biopsy in Detection of Bone Marrow Involvement in Patients with Hodgkin’s or Non-Hodgkin’s Lymphoma?

**DOI:** 10.4274/tjh.2013.0336

**Published:** 2015-08-01

**Authors:** Güven Çetin, M. Ali Çıkrıkçıoğlu, Tuba Özkan, Cumali Karatoprak, M. Cem Ar, Ahmet Emre Eşkazan, Mesut Ayer, Abdullah Cerit, Kübra Gözübenli, Betül Börkü Uysal, Simge Erdem, Nurhan Ergül, Gamze Tatar, T. Fikret Çermik

**Affiliations:** 1 Bezmialem Vakıf University Faculty of Medicine, Department of Internal Medicine, Division of Hematology, İstanbul, Turkey; 2 Bezmialem Vakıf University Faculty of Medicine, Department of General Internal Medicine, İstanbul, Turkey; 3 İstanbul University Cerrahpaşa Faculty of Medicine, Department of General Internal Medicine, Division of Hematology, İstanbul, Turkey; 4 İstanbul Training and Research Hospital, Clinic of Hematology, İstanbul, Turkey; 5 İstanbul Training and Research Hospital, Clinic of Internal Medicine, İstanbul, Turkey; 6 İstanbul Training and Research Hospital, Clinic of Nuclear Medicine, İstanbul, Turkey

**Keywords:** Positron emission tomography, computed tomography, Bone marrow biopsy, Hodgkin’s lymphoma, non-Hodgkin’s lymphoma

## Abstract

**Objective:**

Positron emission tomography and computed tomography (PET/CT) has become an important part of staging and treatment evaluation algorithms of lymphoma. We aimed to compare the results of PET/CT with bone marrow biopsy (BMB) with respect to bone marrow involvement (BMI) in patients with Hodgkin’s lymphoma (HL) and aggressive non-Hodgkin’s lymphoma (aNHL).

**Materials and Methods:**

The medical files of a total of 297 patients diagnosed with HL or aNHL and followed at the hematology clinics of 3 major hospitals in İstanbul between 2008 and 2012 were screened retrospectively and 161 patients with classical HL and aNHL were included in the study. The patients were referred for PET/CT and BMB at the initial staging. BMB was performed as the reference standard for the evaluation of BMI.

**Results:**

There were 61 (38%) HL and 100 (62%) aNHL patients. Concordant results were revealed between PET/CT and BMB in 126 patients (78%) (52 HL, 74 aNHL), 20 with positive PET/CT and BMB results and 106 with negative PET/CT and BMB results. There were discordant results in 35 patients (9 HL, 26 aNHL), 16 of them with positive BMB and negative PET/CT results and 19 of them with negative BMB and positive PET/CT results.

**Conclusion:**

We observed that PET/CT is effective to detect BMI, despite it alone not being sufficient to evaluate BMI in HL and aNHL. Bone marrow trephine biopsy and PET/CT should be considered as mutually complementary methods for detection of BMI in patients with lymphoma. In suspected focal involvement, combining biopsy and PET/CT might improve staging results.

## Abstract (tr)

**Amaç:**

Positron emisyon tomografi ve bilgisayarlı tomografi (PET/BT) lenfomanın tedavi ve evreleme algoritmasının önemli bir parçası oldu. Biz çalışmamız da agresif seyirli non Hodgkin lenfoma (aNHL) ve Hodgkin lenfomalı (HL) hastalarda kemik iliği tutulumunu (KİT) göstermede kemik iliği biyopsisi (KİB) ile PET/BT sonuçlarını karşılaştırmayı amaçladık.

**Gereç ve Yöntemler:**

Çalışmaya 2008-2012 yılları arasında İstanbul’daki 3 büyük hastanede HL ve aNHL tanısı konulan 297 hasta belirlendi. Bu hastaların dosyası retrospektif olarak tarandı ve çalışma kriterlerine uygun 161 hasta çalışmaya alındı. Hastaların başlangıç evrelemesi için PET/BT ve KİB yapıldı. KİB, KİT değerlendirmede standart referans olarak kabul edildi.

**Bulgular:**

Hastaların 61’inde (%38) HL ve 100’ünde (%62) aNHL vardı. Değerlendirilen 126 hastada (%78) (52 HL, 74 aNHL) PET/BT ve KİB arasında uyumlu sonuçlar olduğu görüldü. Bu hastalardan 20’sinde KİT bakımından PET/CT ve KİB sonuçları pozitif ve 106 hastanın PET/CT ve KİB negatif bulundu. Diğer taraftan 35 hastada (9 HL, 26 aNHL) uyumsuzluk vardı. Bu hastaların da 16’sında KİB pozitif iken PET/BT sonuçları negatif bulundu. Geri kalan 19 hastada KİB negatif ve PET/BT sonuçları pozitif saptandı.

**Sonuç:**

Biz aNHL ve HL hastalarını değerlendirmede tek başına yeterli olmamasına rağmen, PET/BT’nin kemik iliği infiltrasyonunu belirlemede etkili olduğunu gözlemledik. Lenfomalı hastalarda kemik iliği infiltrasyonunu belirlemede; KİB ve PET/BT birbirinin tamamlayıcısı olarak düşünülmelidir. Fokal tutulumdan şüphelenildiğinde, KİB ve PET/BT’nin birlikte yapılması hastalığın evrelemesinde daha doğru sonuçlar verebilir.

## INTRODUCTION

Hodgkin’s lymphoma (HL) and aggressive non-Hodgkin’s lymphoma (aNHL) are malignant diseases of the lymphoreticular system. The modality and the duration of treatment in lymphomas are associated with the stage of the disease. In this regard, bone marrow involvement (BMI) plays a crucial role in staging [1,2].

The method considered as the gold standard today in detection of BMI is the trephine bone marrow biopsy (BMB) [3,4]. This method is more useful for diffuse patterns of bone marrow infiltration without intervening areas of normal marrow. However, the invasive nature of this procedure makes its use difficult in patients with poor general condition or tendency to bleed. Moreover, in cases of focal bone marrow infiltration with intervening areas of preserved marrow, lymphoma cells might not be detected at the point of biopsy, leading to false negative results. In this context, a diagnostic tool that eliminates the requirement of an invasive procedure and is able to visualize focal infiltration would facilitate staging. Previous studies have indicated that positron emission tomography and computed tomography (PET/CT) is a convenient method for bone marrow assessment in patients with lymphoma [1,5,6]. In this study, we aimed to compare the results of PET/CT with BMB regarding BMI in patients with HL and aNHL.

## MATERIALS AND METHODS

The medical files of a total of 297 patients diagnosed with HL or aNHL and followed at the hematology clinics of 3 major hospitals in İstanbul between 2008 and 2012 were screened retrospectively and 161 patients with classical HL and aNHL were included in the study. Patients with indolent aNHL, chronic lymphocytic leukemia/small lymphocytic lymphoma, or nodular lymphocyte-predominant HL and those with secondary malignancies were excluded from the study. Written permission was obtained from the medical directors of the relevant hospitals for retrospective file screening. The study was approved by the Ethics Committee of the Bezmialem Vakıf University Medical Faculty.

All BMB samples were obtained from the dorsal iliac crest (unilateral), with a length of 10-15 mm. They were fixed in Hollande’s solution and sent to the pathology department for histological evaluation. The BMB samples were reviewed by a hematopathologist and BMB was considered the reference standard to detect BMI. PET/CT imaging was performed using a Siemens Biograph LSO HI-REZ integrated PET/CT camera (Biograph 6, Siemens Medical Solutions, Chicago, IL, USA). PET/CT scans were obtained 60-80 min after the administration of 5.4 MBq/kg 18F-fluorodeoxyglucose (FDG). The patients fasted for at least 6 h and serum glucose levels were below 120 mg/dL in all patients. All PET/CT images were visually assessed for BMI by 2 experienced nuclear medicine physicians without the results of the BMBs at the Department of Nuclear Medicine of the İstanbul Training and Research Hospital. The uptake of FDG in the bone marrow was visually classified into 3 categories: (1) diffusely intense FDG uptake in the bone marrow, which represents diffuse involvement; (2) lesions with focal intense FDG uptake, which were suggestive of focal involvement of bone marrow; (3) no focal or diffuse increased FDG uptake in bone marrow, which represents normal bone marrow functions.

Anemia, infection, or other pathological situations that could cause false positivity on PET/CT imaging were excluded by a physical examination, complete blood cell counts, biochemistry tests including acute phase reactant tests, serology tests, pulmonary function tests, echocardiography, and CT of the chest, abdomen, and pelvis. Positive PET/CT results were defined as a malignancy by nuclear medicine physicians.

### Statistical Analysis

Numerical values are given as means and standard deviations; nominal values were compared by using kappa statistics. Sensitivity, specificity, negative and positive predictive values, and overall accuracy values of PET/CT were calculated as compared with the gold standard. Two-tailed p-values of less than 0.05 were considered statistically significant.

## RESULTS

**Figure 1:**
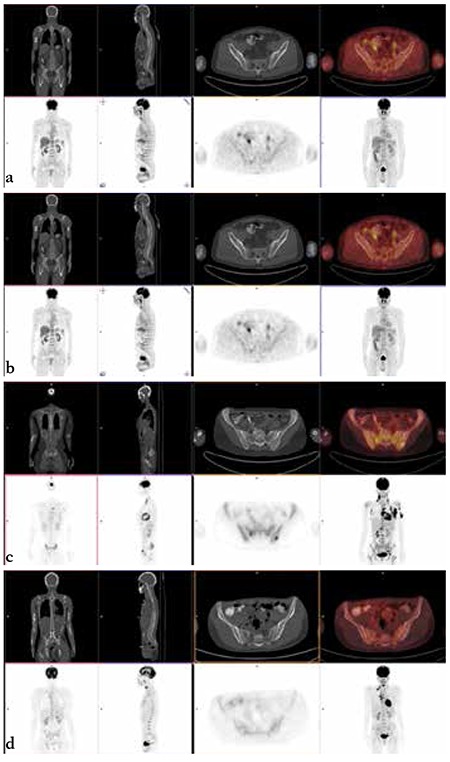
Positron emission tomography and computed tomography images. a) Aggressive non-Hodgkin’s lymphoma patient bone marrow involvement-positive in bone marrow biopsy and bone marrow involvement-negative in positron emission tomography and computed tomography imaging, b) Aggressive non-Hodgkin’s lymphoma patient bone marrow involvement-negative in bone marrow biopsy and bone marrow involvement-positive in positron emission tomography and computed tomography imaging, c) Hodgkin’s lymphoma patient bone marrow involvement-positive in bone marrow biopsy and positron emission tomography and computed tomography imaging together, d) Hodgkin’s lymphoma patient bone marrow involvement-negative in bone marrow biopsy and positron emission tomography and computed tomography imaging together.

There were 61 (37.8%) and 100 (62.2%) patients with HL and aNHL, respectively. Table 1 summarizes patient characteristics, including age, sex, and stages of disease. BMI was detected in 36 patients (22.3%) by BMB and 39 patients (24.2%) by PET/CT. If BMB and PET/CT results supported each other, they were considered as concordant results. Regarding the type of lymphoma, this study showed a concordance between PET/CT and BMB in 126 patients (78%) (74 aNHL, 52 HL), of which 20 were positive (15 aNHL, 5 HL) and 106 were negative (59 aNHL, 47 HL). If BMB and PET/CT results conflicted with each other, they were considered as discordant results. Discordant results were observed in 35 cases (22%): 19 of them with positive PET/CT and negative BMB results (12 aNHL, 7 HL) and 16 with negative PET/CT and positive BMB results (14 aNHL, 2 HL). Comparison of BMB and PET/CT results in the detection of BMI are summarized in Table 2 (Figure 1).

In patients with HL, a statistically significant accordance was seen between BMB and PET/CT with respect to BMI (kappa value 0.446; p<0.001). The nonrandom concordance rate between the 2 methods was 44.6%. When we considered BMB as the gold standard, the sensitivity and specificity of PET/CT in HL cases were 71.4% and 87%, respectively. Positive and negative predictive values were 41.7% and 95%, respectively. Overall accuracy was 85.2%.

The accordance between BMB and PET with respect to BMI in aNHL was also found to be significant (kappa value 0.355; p<0.001). The nonrandom concordance ratio between the 2 methods was 35.5%. When we utilized BMB as the gold standard, the sensitivity and specificity of PET/CT in aNHL were 51.7% and 83.0%, respectively. Positive and negative predictive values were 55.5% and 80.8%, respectively. Overall accuracy was 74%.

We found a statistically significant compliance between results of BMI and PET/CT with regard to BMI in all patients (kappa value 0.392, p<0.001). A nonrandom concordance of 39.2% between the 2 methods was observed. According to BMB as the gold standard, the sensitivity and specificity of PET/CT were 55.6% and 84.8%, respectively. According to BMB, positive predictive values, negative predictive values, and general accuracy of PET/CT were 51.3%, 86.9%, and 78.3%, respectively.

## DISCUSSION

Disease staging is required to determine treatment strategies in patients with HL and aNHL. Presence or absence of BMI plays a determinative role in staging [1,4]. The gold standard in detecting BMI is the trephine BMB. However, since it is an invasive procedure and may give false negative results in cases of focal involvement, the effectiveness of noninvasive methods that are able to detect focal involvements, such as PET/CT, has also been considered [7,8].

PET/CT imaging has become an important part of lymphoma staging and treatment algorithms [7,8,9]. Moreover, the presence of persistent FDG uptake during or after chemotherapy has a high sensitivity and specificity for predicting disease recurrence [9].

Staging is a crucial step in the initial diagnostic work-up of lymphoma with the aim of determining the extent of the disease and detecting subclinical disease. Early studies revealed strong FDG involvement in the initial staging of both HL and aNHL [10,11,12]. Bangerter et al. found occult disease in 5 patients (11%) in addition to PET/CT positivity detected in 38/44 (86%) of patients with proven disease [11]. PET/CT was able to detect 128 abnormal regions, of which 11 could not be detected previously by conventional methods. PET/CT has changed the management of the disease by 14% [9]. In a prospective study by Young et al., biopsies were taken from the sites of involvement detected by PET/CT to confirm the infiltration, and it was demonstrated that PET/CT changed the disease stage in 59% of the 45 patients included in the study [13].

In our study we evaluated PET/CT as a substitute for BMB. According to our data, PET/CT detected 20 positive cases that were detected with BMB. Additionally, 19 (11.8%) cases that were considered false-positive according to BMB were evaluated for staging by PET/CT. At this point we accepted modified stages at the beginning of the chemotherapy. By the time biopsy results were revealed, we had started chemotherapy. We correlated these results with focal involvement. During chemotherapy we could not retake the biopsy due to it not already being in the routine practice. Nineteen discordant cases were treated with chemotherapy because bone marrow infiltration could not be excluded. The results of 16 discordant cases showed false negativity of PET/CT imaging.

One of the issues in the staging of aNHL is detection of BMI [14]. Many studies have shown that PET/CT is superior to conventional imaging in evaluation of bone marrow infiltration in conjunction with histological verification [15,16]. In a study that included 50 patients (12 with HL and 38 with aNHL), an overall accuracy of 93% was attained [17]. In another study that compared 78 patients (39 with HL and 39 with aNHL), although BMI was found in 13% of the patients by PET/CT, false negativity of 5% was found. When PET/CT and BMB were compared, overall accuracy was 95% for PET/CT and 89% for BMB [8]. In a study performed by Muslimani et al., 97 patients with aNHL were studied. Whole-body PET/CT and unilateral iliac BMB were performed in the patients for initial staging, and as a result, sensitivity and specificity of PET/CT in detection of bone marrow infiltration in the patients were found to be 79% and 91%, respectively [18]. According to the study conducted by Purz et al. in pediatric patients with HL, while BMB was positive in 7 of 175 patients, BMI was detected by PET/CT in 45 patients. Moreover, a typical multifocal pattern was found in 32 of these 45 patients and involvements detected by PET/CT were reported to disappear after chemotherapy [19].

However, in a study performed by Jerusalem et al., which included 42 patients, accuracy of detection by PET/CT was 39% in indolent NHL cases [20]. This appears to be a result of low FDG uptake in some subtypes of lymphoma and of the difficulty in discriminating the infiltration pattern from physiological bone marrow uptake [21]. In a prospective study of 52 patients that compared BMB with PET/CT and CT, PET/CT was able to visualize bone marrow infiltration more accurately than CT (p<0.05) and at a comparable level to bone marrow trephine biopsy. PET/CT led to treatment changes in 8% of patients [22]. In a review performed by Haioun et al., results obtained by using PET/CT alone were in line with conventional imaging and BMB in only 80% of the cases. Using PET/CT alone yielded superior results to both methods in 8% of the cases and inferior results to both of them in 12 of the cases [23]. In a metaanalysis of 587 patients that evaluated PET/CT results in detection of bone marrow involvement in HL and aNHL, this method was designated as a good, but not excellent, tool [24].

PET/CT is the best noninvasive imaging technique in the evaluation of the response to treatment [25]. Early studies have shown that persistence of FDG uptake after treatment was associated with high recurrence rates [26,27]. Similar results have been reported by others [28,29,30]. However, it should be considered that increased FDG uptake may also be related to active infection and inflammation. Before any treatment decision is made, PET/CT images should be in line with the clinical findings, other imaging techniques, and/or biopsy [31]. Negative PET/CT imaging cannot rule out minimal residual disease that may lead to clinical relapse [32]. Currently, PET/CT is widely used for the evaluation of response after the completion of treatment. To a lesser extent, it is used for staging before treatment. PET/CT cannot be a substitute for CT or BMB in staging before treatment, but it provides complementary information to them. It is much better than CT in conclusive evaluation of response to treatment, because this method can discriminate living tumor tissue from fibrosis and necrosis. The predictive potential of PET/CT imaging in the follow-up of patients with lymphoma in the absence of clinical, biochemical, or radiographic evidence of disease is unknown. Large prospective studies are needed to assess the role of PET/CT imaging with regard to patient follow-up and costs [33].

In conclusion, bone marrow trephine biopsy and PET/CT should be considered as mutually complementary methods for detection of BMI in patients with lymphoma. In cases of suspected focal involvement, combining biopsy and PET/CT might improve staging results. The current literature suggests that PET/CT may be more sensitive than BMB in cases with focal involvements. Our results partly confirm the literature data.

## Figures and Tables

**Table 1:**
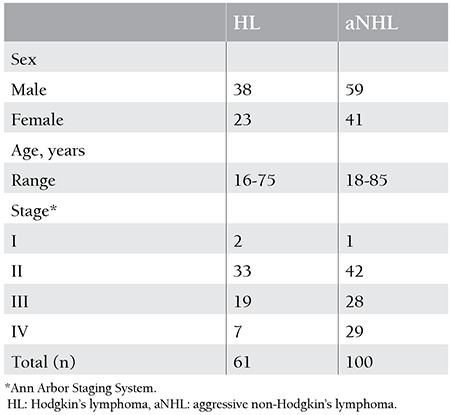
Patient characteristics.

**Table 2:**
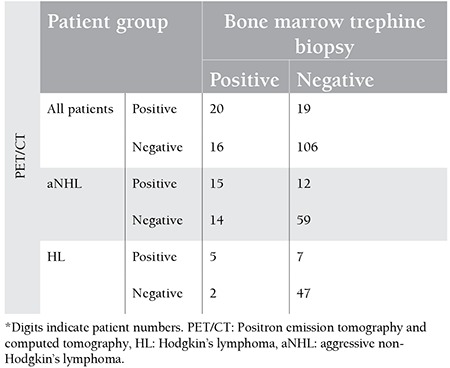
Cross-table for patients with bone marrow involvement (positron emission tomography and computed tomography vs. trephine biopsy).*
